# Cognitive profiles discriminate between genetic variants of behavioral frontotemporal dementia

**DOI:** 10.1007/s00415-020-09738-y

**Published:** 2020-02-12

**Authors:** J. M. Poos, L. C. Jiskoot, S. M. J. Leijdesdorff, H. Seelaar, J. L. Panman, E. L. van der Ende, M. O. Mol, L. H. H. Meeter, Y. A. L. Pijnenburg, L. Donker Kaat, F. J. de Jong, J. C. van Swieten, J. M. Papma, E. van den Berg

**Affiliations:** 1 Department of Neurology, Alzheimer Center, Erasmus MC University Medical Center, Dr. Molewaterplein 40, 3000 CA Rotterdam, The Netherlands; 2 Department of Radiology, Leiden University Medical Center, Leiden, The Netherlands; 3 Dementia Research Center, University College London, London, UK; 4 Department of Psychiatry and Psychology, Maastricht University, Maastricht, The Netherlands; 5 Department of Neurology, Alzheimer Center, Location VU University Medical CenterAmsterdam Neuroscience, Amsterdam University Medical Center, Amsterdam, The Netherlands; 6 Department of Clinical Genetics, Erasmus MC University Medical Center, Rotterdam, The Netherlands

**Keywords:** Frontotemporal dementia, FTD, Genetic, Neuropsychology, Cognition

## Abstract

**Introduction:**

Trials to test disease-modifying treatments for frontotemporal dementia are eagerly awaited and sensitive instruments to assess potential treatment effects are increasingly urgent, yet lacking thus far. We aimed to identify gene-specific instruments assessing clinical onset and disease progression by comparing cognitive functioning between bvFTD patients across genetic mutations.

**Methods:**

We examined differences in 7 cognitive domains between bvFTD patients with *GRN* (*n* = 20), *MAPT* (*n* = 29) or *C9orf72* (*n* = 31) mutations, and non-carriers (*n* = 24), and described longitudinal (*M* = 22.6 months, SD = 16.6) data in a subsample (*n* = 27).

**Results:**

Patients showed overall cognitive impairment, except memory recall, working memory and visuoconstruction. *GRN* patients performed lower on executive function (mean difference − 2.1; 95%CI − 4.1 to − 0.5) compared to *MAPT* and lower on attention compared to *MAPT* (mean difference − 2.5; 95%CI − 4.7 to − 0.3) and *C9orf72* (mean difference − 2.4; 95%CI − 4.5 to − 0.3). Only *MAPT* patients were impaired on delayed recall (mean difference − 1.4; 95%CI − 2.1 to − 0.7). *GRN* patients declined rapidly on attention and memory, *MAPT* declined in confrontation naming, whereas *C9orf72* patients were globally impaired but remained relatively stable over time on all cognitive domains.

**Discussion:**

This study shows gene-specific cognitive profiles in bvFTD, which underlines the value of neuropsychological tests as outcome measures in upcoming trials for genetic bvFTD.

**Electronic supplementary material:**

The online version of this article (10.1007/s00415-020-09738-y) contains supplementary material, which is available to authorized users.

## Background

Frontotemporal dementia (FTD) includes a large spectrum of neurodegenerative disorders with a variable clinical presentation of either progressive behavioral and executive deficits (behavioral variant FTD [bvFTD]) or language dysfunction (primary progressive aphasia [PPA]), associated with prominent frontal and/or anterior temporal lobe degeneration [29]. bvFTD is the most common phenotype in the clinical spectrum and the neuropsychological profile is generally characterized by impaired executive function (e.g., planning, set shifting and working memory), social cognition (e.g., theory of mind, emotional processing), whereas memory and visuoconstruction are relatively spared in comparison to executive dysfunction [27]. However, it is becoming increasingly clear that these cognitive impairments vary in severity and progression. Executive dysfunction may be absent or overshadowed by behavioral dysfunctions and/or significant episodic memory impairment can be present even at the earliest stages of the disease [13, 29]. Factors influencing the variety in cognitive impairments between patients with bvFTD are not yet understood.

In 20–30% of cases, FTD has an autosomal dominant pattern of inheritance (i.e., mutations in microtubule-associated protein tau [*MAPT*], progranulin [*GRN*] genes, or a repeat expansion in chromosome 9 open reading frame 72 [*C9orf72*] gene) [20]. *GRN* mutations often lead to a prominent asymmetrical pattern of atrophy in the frontal, temporal and parietal lobes, and are associated with behavioral deficits, apraxia and language disorders, most frequently resulting in a clinical diagnosis of bvFTD or non-fluent variant PPA (nfvPPA) and is often accompanied by parkinsonism [21, 29]. *MAPT* mutations show localized temporal lobe involvement associated with behavioral and semantic deficits, resulting in bvFTD as the main phenotype, and is occasionally accompanied by a parkinson-dominant phenotype with corticobasal syndrome (CBS) or progressive supranuclear palsy (PSP) syndrome [4, 29]. The atrophy associated with *C9orf72* repeat expansion is rather diffuse, and as a result leads to a more widespread pattern of clinical and cognitive features such as behavioral and executive impairment but also notable psychiatric features including psychosis and anxiety [1, 24]. This is usually accompanied by a clinical diagnosis of bvFTD and/or motor neuron disease (MND) [29]. Cognitive differences between genetic variants of FTD can, in part, be explained by the associated phenotypes (i.e., bvFTD or PPA). Yet, there is also a high variability in the profile of cognitive decline between patients with bvFTD. This might be due to the different atrophy patterns associated with each genetic mutation.

Implementation of clinical trials to test disease-modifying treatments for bvFTD is eagerly awaited and instruments that can signal clinical onset and measure potential longitudinal treatment effects are increasingly urgent. Although a small number of studies have presented comprehensive clinical descriptions of FTD patients with mutations in *MAPT*, *GRN* or *C9orf72 *[21, 24, 26, 33, 38], there are even less studies that concisely and elaborately describe the specific cognitive profiles associated with each mutation or make direct comparisons between genetic variants. Investigating the distinct cognitive profiles between genetic variants of bvFTD will enable us to identify gene-specific sensitive cognitive outcome measures for signaling disease onset, tracking disease progression and measuring potential treatment effects in upcoming therapeutic trials.

We compared cognitive profiles cross-sectional in patients with bvFTD due to mutations in *GRN*, *MAPT* or *C9orf72* and report patterns of cognitive decline in a subset of patients with follow-up data.

## Methods

### Participants

Patients were included in an ongoing genetic-epidemiological study, after referral to the outpatient clinic of the Erasmus Medical Center between 1994 and 2018. We reviewed data of patients with a known pathogenic mutation in *MAPT* or *GRN*, or repeat expansion in *C9orf72*, who had a clinical diagnosis of bvFTD and underwent one or multiple neuropsychological assessments (*n* = 81) [27]. Standardized work up consisted of a neurological and neuropsychological assessment, laboratory testing and brain imaging. Diagnosis was determined in a multidisciplinary consensus meeting of the FTD Expertise Center of the Erasmus MC University Medical Center, involving experienced neurologists, neuropsychologists, neuroradiologists, geriatricians, and a care consultant according to established diagnostic criteria for bvFTD [27]. Patients were categorized into three subtypes based on their clinical presentation; disinhibited (e.g., loss of social manners, inappropriate and impulsive behavior), apathetic (e.g., lack of interests in life activities and/or interactions with others, little motivation to undertake action) and stereotypic (e.g., pacing, picking, ritualistic behavior) [32]. For a separate analysis, patients with a *GRN* mutation were divided based on predominant left-sided (*n* = 10), right-sided (*n* = 4) or generalized atrophy (*n* = 4) as described in the report of the radiologist. For two patients there was no report available. Twenty-four non-carrier participants that were part of an ongoing epidemiological study of Dutch pathologically confirmed genetic FTD families [FTD Risk cohort (FTD-RisC [8])], were used as a reference (matched for age, education and sex).

### Neuropsychological assessment

As the standardized neuropsychological test battery underwent some changes over the time period of 24 years, the protocol differed between patients. We only included tests with ten or more subjects in each group. Global cognitive functioning was screened with the Mini-Mental State Examination (MMSE) [10] and the Frontal Assessment Battery (FAB) [9]. For executive functioning we used the Trail making Test (TMT) part B [7], Stroop Color Word Test (SCWT) interference card III [17], Modified Wisconsin Card Sorting Test (mWCST) [25], and Similarities of the Wechsler Adult Intelligence Scale III-NL (WAIS-III) [39]. For attention and concentration we used TMT part A [7], and the SCWT word reading (I) and color naming card (II) [17]. For language we used the Boston Naming Test (BNT) [18], and semantic and letter fluency. For episodic memory—immediate recall, we used the Rey Auditory Verbal Learning Test (RAVLT) Dutch version [37]—immediate recall trial, the Rivermead Behavioral Memory Test (RBMT) [41] Dutch version – immediate recall, and the short version of the Visual Association Test (VAT) [23]. For episodic memory—delayed recall, we used the RAVLT Dutch version—delayed recall trial and the RBMT Dutch version—delayed recall trial. For working memory we used the total score of the WAIS-III Digit Span (forward upper limit 9; backward upper limit 8) [39]. For visuoconstruction we used the Clock Drawing test [30]. For the BNT, the VAT and Clock Drawing Test different test versions were used (respectively, 15-item/30-item/60-item, 12-item/24-item, 3-item/14-item). For these respective tests, the scores were extrapolated to match performance on the version with the maximum score. The TMT and SCWT scores were truncated to 300 s for patients that exceeded the time limit or were unable to complete the test. The mean was calculated for SCWT cards I and II, as both tests are measures of attention/processing speed. When patients underwent multiple neuropsychological assessments in a short period of time (≤ 4 months) we considered this as one baseline assessment (*n* = 3); for tests that were performed at both assessments, the score of the first assessment was included in the cross-sectional baseline analyses.

### Statistical analyses

Statistical analyses were performed using SPSS Statistics 21.0 (IBM Corp., Armonk, NY). To aid interpretation, we standardized all raw neuropsychological test scores by converting them into z-scores (i.e., individual test score minus the mean of non-carriers, divided by the standard deviation (SD) of non-carriers). Composite domain scores constituted the mean of the z-scores for the tests within one domain (as described in Sect. 2.2). When a neuropsychological test was missing, the domain was calculated based on the remaining test scores in that specific domain. On TMT A and B, SCWT card I + II and card III, WCST, and VAT, a log10 transformation was applied to normalize the data. We set the significance level at *p* < 0.05 (2-tailed) across all comparisons. We compared demographic data with one-way analyses of variance. We analyzed sex and subtype differences between groups using Pearson *χ*^2^ tests. Neuropsychological data between groups were analyzed by means of one-way analysis of covariance. For the comparison of each mutation carrier group to non-carriers we used age as a covariate, and performed planned contrasts between each mutation carrier group and non-carriers. We compared mutation carrier groups in pairwise comparisons with disease duration as an additional covariate. Additional analyses were performed to compare cognitive domains in *GRN* patients with a predominant left-sided, right-sided or generalized atrophy pattern. All post-hoc analyses were Bonferroni corrected for multiple comparisons. Effect sizes (Cohen’s *d*) were calculated for the (significant) differences in test scores. According to Cohen’s Nomenclature [6] *d* > 0.80 indicates a large difference. A bias-corrected 95% confidence interval (CI) was calculated based on the standard error. The percentage overlap (%OL) in (significant) test scores between groups was also reported according to Zakzanis’ calculations [42]; *d* = 0 equates to 100% overlap, *d* = 1.0 equates to 45% overlap and *d* = 3 equates to less than 5% overlap in group scores. In addition, we report a description of a subset of patients with longitudinal data both on composite cognitive domains and neuropsychological tests (as described in Sect. 2.2). Due to the small sample size, we did not perform longitudinal statistical analysis.

## Results

### Demographics

Demographic data are shown in Table 1. *MAPT* mutation carriers were significantly younger than the other mutation carrier groups. *C9orf72* repeat expansion carriers were older and had a significantly longer disease duration than the other mutation carrier groups. *GRN* mutation carriers performed significantly lower on MMSE and FAB.

**Table 1:** Demographic features

	*MAPT* mutation carriers (*n* = 29)	*GRN* mutation carriers (*n* = 20)	*C9orf72* mutation carriers (*n* = 31)	Non-carriers (*n* = 24)	*p* Value	Group differences
Age at baseline, y	52.6 ± 5.5	60.4 ± 7.4	62.1 ± 9.1	56.1 ± 5.7	< 0.01	*MAPT* < *GRN* = *C9orf72* NC < *C9orf72*
Sex (% female)	10 (34.5%)	12 (57.1%)	13 (41.9%)	11 (45.8%)	0.6	n.s
Educational level ^a^ (median (IQR))	5 (2)	5 (2)	5 (2)	5 (0)	0.8	n.s
Disease duration, y	1.4 ± 2.0	1.0 ± 1.1	3.1 ± 2.7	NA	< 0.01	*MAPT* = *GRN* < *C9orf72*
Subtype dis—apa—ster	9—15—5	6—14—0	6—21—3	NA	0.3	n.s
MMSE	25.9 ± 2.9	22.5 ± 6.3	26.5 ± 2.7	29.3 ± 0.8	< 0.01	*GRN* < *MAPT* < NC *GRN* < *C9orf72*
FAB	14.7 ± 3.2	10.0 ± 4.7	13.9 ± 3.4	16.1 ± 1.7	< 0.01	*GRN* < *MAPT* = NC

Values indicate mean ± SD or n (%) unless otherwise specified

*MAPT* microtubule-associated protein tau, *GRN* progranulin, *C9orf72* chromosome 9 open reading frame 72, *NC* non-carriers, *dis* disinhibited, *apa* apathetic, *ster* stereotypic, *MMSE* Mini-Mental State Examination, *FAB* Frontal Assessment Battery, *n.s* not significant

^a^Verhage Dutch educational system categorized into levels from 1 = less than 6 years of primary education to 7 = academic schooling

### Cross-sectional analysis—comparison to non-carriers

Table 2 shows the baseline *z*-scores of neuropsychological tests for the three mutation carrier groups. Compared to non-carriers, all mutation carrier groups were significantly impaired on language, attention/mental processing speed and executive functioning, but not on working memory and visuoconstruction. Executive functioning was most sensitive to differentiate *GRN* mutation carriers from non-carriers (mean difference − 5.1; 95%CI − 6.5 to 3.7, *p* < 0.01, *d* = 2.9, %OL = 8.8–7.2), whereas language was most sensitive to differentiate *C9orf72* (mean difference − 2.1; 95%CI − 2.8 to − 1.3, *p* < 0.01, *d* = 2.0, %OL = 18.9) and *MAPT* mutation carriers (mean difference − 2.3; 95%CI − 3.0 to − 1.6, *p* < 0.01, *d* = 1.8, %OL = 22.6) from non-carriers. On neuropsychological test level this translated into logWCST being most sensitive to differentiate *GRN* mutation carriers from non-carriers (mean difference − 1.0; 95%CI − 1.4 to − 0.7, *p* < 0.01, *d* = 3.0, %OL = 7.2), RBMT direct (mean difference − 2.0; 95%CI − 2.9 to − 1.2, *p* < 0.01, *d* = 2.4, %OL = 13) and delayed (mean difference − 2.2; 95%CI − 3.1 to − 1.3, *p* < 0.01, *d* = 2.4, %OL = 13) recall were most sensitive to differentiate *MAPT* mutation carriers from non-carriers, and logSCWT I and II was most sensitive to differentiate *C9orf72* mutation carriers from non-carriers (mean difference 0.34; 95%CI 0.2–0.5, *p* < 0.01, *d* = 2.4, %OL = 13). Concerning memory, *GRN* (mean difference − 4.5; 95%CI − 7.6 to − 1.3, *p* = 0.02, *d* = 1.1, %OL = 41.1) and *MAPT* (mean difference − 3.8; 95%CI − 6.8 to − 0.8, *p* = 0.04, *d* = 0.7, %OL = 57) mutation carriers were equally impaired in immediate recall, also with significant impairment in delayed recall in the latter group (mean difference − 1.4; 95%CI − 2.1 to − 0.7, *p* < 0.01, *d* = 1.2, %OL = 37.8). Analyses showed that *C9orf72* repeat expansion (mean difference − 1.2; 95%CI − 2.0 to − 0.4, *p* = 0.01, *d* = 1.4, %OL = 31.9) and *MAPT* mutation (mean difference − 1.2; 95%CI − 2.0 to − 0.5, *p* < 0.01, *d* = 1.0, %OL = 44.6) carriers were equally impaired on RAVLT—immediate recall, but in addition with significant impairment on RAVLT—delayed recall in the latter group (mean difference − 1.1; 95%CI − 1.8 to − 0.3, *p* = 0.02, *d* = 0.8, %OL = 52.6). *GRN* mutation carriers were only significantly impaired on the VAT (mean difference − 0.5; 95% CI − 0.8 to − 0.1, *p* = 0.02, *d* = 0.7, %OL = 57).

**Table 2:** Differences between genetic mutation carrier groups on neuropsychological tests within seven cognitive domains

Domain	*MAPT* mutation carriers	*n*	*GRN* mutation carriers	*n*	*C9orf72* mutation carriers	*n*	*p* Value	Group differences
Language	− 2.2 ± 1.5	26	− 2.5 ± 1.4	20	− 2.3 ± 1.4	28	< 0.01	*GRN* = *MAPT* = *C9orf72* < NC
BNT60	− 2.4 ± 2.6	23	− 2.3 ± 2.5	17	− 2.1 ± 1.9	22	< 0.01	*GRN* = *MAPT* = *C9orf72* < NC
Semantic fluency	− 2.5 ± 1.2	26	− 2.7 ± 1.3	20	− 2.6 ± 1.3	27	< 0.01	*GRN* = *MAPT* = *C9orf72* < NC
Letter fluency	− 0.6 ± 1.3	17	− 2.2 ± 0.6	13	− 1.2 ± 0.8	18	< 0.01	*GRN* < *MAPT* = *C9orf72* < NC
Attention and mental processing speed	− 1.2 ± 1.9	25	− 4.3 ± 4.1	18	− 2.1 ± 2.3	24	< 0.01	*GRN* < *MAPT* = *C9orf72* < NC
TMT A*	− 0.8 ± 1.2	25	− 3.9 ± 4.1	18	− 2.3 ± 3.6	24	< 0.01	*GRN* < *MAPT* < NC *C9orf72* < NC
SCWT card I and II*	− 2.7 ± 3.2	21	− 4.9 ± 5.8	17	− 3.0 ± 1.6	19	< 0.01	*GRN* = *MAPT* = *C9orf72* < NC
Executive functioning	− 2.7 ± 2.5	25	− 5.3 ± 2.5	18	− 4.0 ± 2.6	24	< 0.01	*GRN* < *MAPT* < NC *C9orf72* < NC
TMT B*	− 2.3 ± 2.8	24	− 5.5 ± 2.7	18	− 3.8 ± 2.6	23	< 0.01	*GRN* < *MAPT* < NC *C9orf72* < NC
SCWT card III*	− 3.2 ± 4.0	20	− 7.7 ± 4.6	17	− 5.3 ± 3.4	19	< 0.01	*GRN* < *MAPT* < NC *C9orf72* < NC
WCST concepts*	− 1.6 ± 1.6	16	− 2.8 ± 0.6	14	− 1.1 ± 1.6	14	< 0.01	*GRN* < *C9orf72* < NC *MAPT* < NC
WAIS-III Similarities	− 1.6 ± 2.2	11	− 2.8 ± 1.4	10	− 1.8 ± 1.3	10	< 0.01	*GRN* = *C9orf72* = *MAPT* < NC
Memory—learning	− 3.3 ± 6.6	25	− 4.7 ± 6.1	18	− 3.0 ± 5.3	24	0.02	*MAPT* = *GRN* < NC
RAVLT-learning	− 1.1 ± 1.2	21	− 1.1 ± 1.8	14	− 1.4 ± 1.1	19	< 0.01	*MAPT* = *C9orf72* = *GRN* < NC
RBMT-learning	− 2.1 ± 0.7	10	− 2.0 ± 1.0	10	− 1.7 ± 1.0	11	< 0.01	*GRN* = *MAPT* = *C9orf72* < NC
VAT^*^	− 6.3 ± 10.7	12	− 10.6 ± 11.8	12	− 5.0 ± 9.4	16	0.02	*GRN* < NC
Memory—recall	− 1.3 ± 1.3	24	− 1.0 ± 1.6	16	− 0.9 ± 1.2	22	< 0.01	*MAPT* < NC
RAVLT-recall	− 0.9 ± 1.3	21	− 0.7 ± 1.7	14	− 0.5 ± 1.1	19	0.05	*MAPT* < NC
RBMT-recall	− 2.2 ± 0.8	9	− 1.7 ± 1.0	10	− 1.6 ± 1.1	11	< 0.01	*GRN* = *MAPT* = *C9orf72* < NC
Working memory	− 0.4 ± 1.7	11	− 1.1 ± 2.2	8	− 1.2 ± 1.2	10	0.09	n.s
WAIS-III Digit Span	− 0.4 ± 1.7	11	− 1.1 ± 2.2	8	− 1.2 ± 1.2	10	0.09	n.s
Visuoconstruction	− 0.7 ± 2.4	20	− 1.0 ± 1.6	18	− 1.2 ± 2.7	22	0.30	n.s
Clock drawing	− 0.7 ± 2.4	20	− 1.0 ± 1.6	18	− 1.2 ± 2.7	22	0.30	n.s

Values indicate mean ± SD

Non-carriers were excluded as they had means of zero and SDs of one by definition

*MAPT* microtubule-associated protein tau, *GRN* progranulin, *C9orf72* chromosome 9 open reading frame 72, *BNT* Boston Naming Test, *TMT* Trail Making Test, *WCST* Wisconsin Card Sorting Test, *RAVLT* Rey Auditory Verbal Learning Test, *RBMT* Rivermead Behavioral Memory Test, *VAT* Visual Association Test, *n.s* not significant

The *p* values constitute interaction terms of univariate analyses of covariance (corrected for age) (on z-scores and ^*^log10 transformed data)

### Cross-sectional analysis—comparison between mutation carrier groups

On domain level, *GRN* mutation carriers could be differentiated from *MAPT* mutation carriers by significantly lower attention and mental processing speed (mean difference − 2.5; 95%CI − 4.7 to − 0.3, *p* = 0.02, *d* = 1.0, %OL = 44.6), and executive functioning (mean difference − 2.1; 95%CI − 4.1 to − 0.5, *p* = 0.03, *d* = 1.1, %OL = 41.1) (Table 2). On test level, *GRN* mutation carriers performed significantly worse on letter fluency (mean difference − 1.3; 95%CI − 2.2 to − 0.4, *p* < 0.01, *d* = 1.6, %OL = 26.9), TMT A (mean difference − 0.48; 95%CI − 0.1 to − 0.09, *p* = 0.02, *d* = 1.2, %OL = 37.8), TMT B (mean difference − 0.5; 95%CI − 0.1 to − 0.9, *p* = 0.02, *d* = 1.1, %OL = 41.1), and SCWT card III (mean difference -0.4; 95%CI − 0.1 to − 0.8, *p* = 0.01, *d* = 0.7, %OL = 57). *GRN* mutation carriers could be differentiated from *C9orf72* repeat expansion carriers by significant lower attention and mental processing speed (mean difference -2.4; 95%CI − 4.5 to − 0.3, *p* = 0.02, *d* = 0.7, %OL = 57). On test level, *GRN* mutation carriers performed significantly worse on letter fluency (mean difference − 1.1; 95%CI − 2.0 to − 0.3, *p* = 0.01, *d* = 1.3, %OL = 34.7) and WCST (mean difference − 0.6; 95%CI − 1.1 to -0.1, *p* = 0.02, *d* = 1.2, %OL = 37.8) compared to *C9orf72* mutation carriers. The other tests did not differentiate between mutation carrier groups. On domain level, *GRN* patients with predominant left-sided atrophy performed significantly worse on language compared to *GRN* patients with predominant right-sided atrophy (mean difference − 2.3; 95%CI − 4.3 to − 0.3, *p* = 0.02, *d* = 2.3, %OL = 13). There were no other significant differences between *GRN* mutation carriers with different atrophy patterns (see Supplementary material).

### Within-individual longitudinal trajectories of cognitive decline

**Fig. 1:**
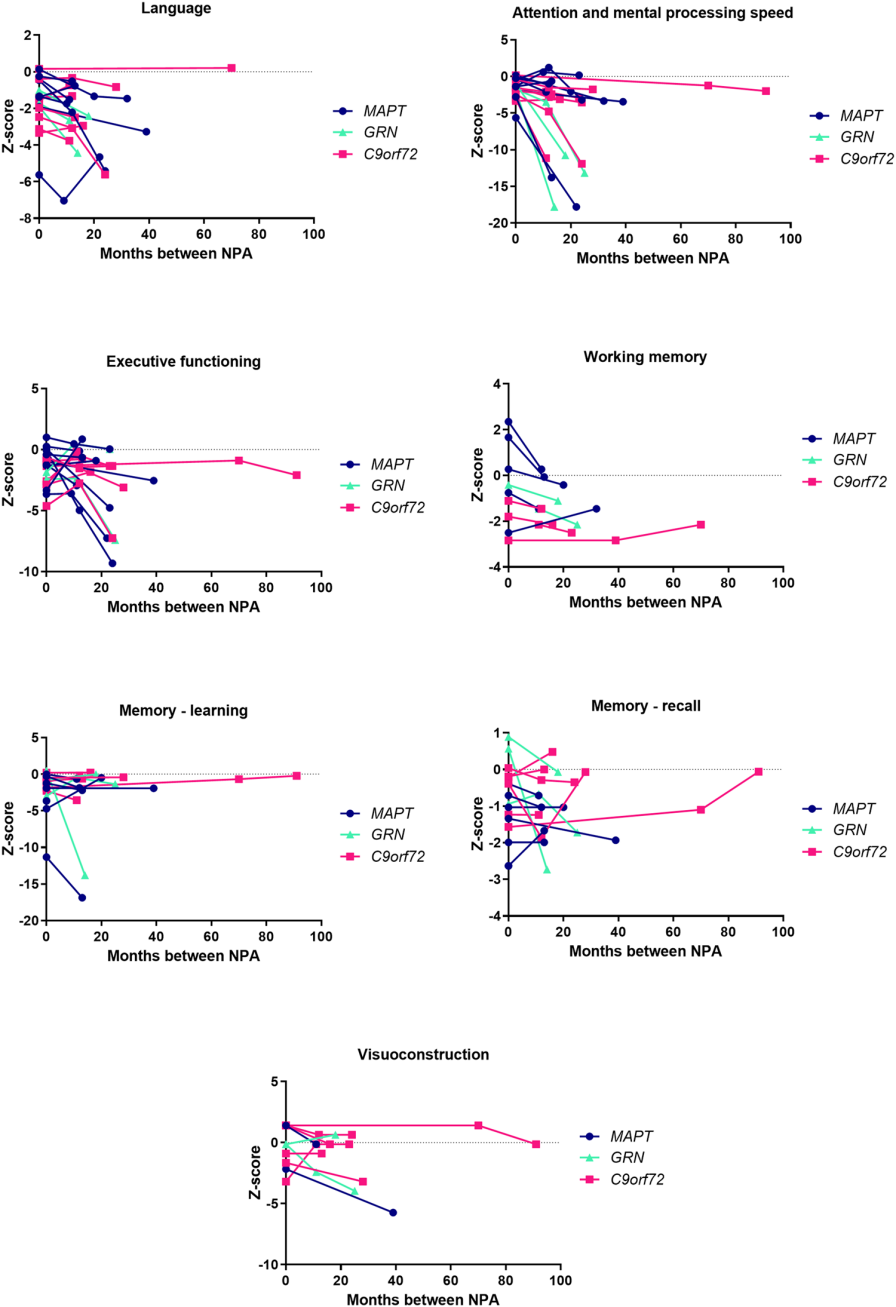
Within-individual trajectories of cognitive decline on seven cognitive domains. *NPA* neuropsychological assessment, *MAPT* microtubule-associated protein tau, *GRN* progranulin, *C9orf72* chromosome 9 open reading frame 72. Raw data for each neuropsychological test were first converted to z-scores by standardization to the baseline data of non-carriers. Composite cognitive domain scores were calculated. Each subplot presents the trajectory on a specific cognitive domain. Data are available in: language (*MAPTn* = 9; *GRNn* = 3; *C9orf72n* = 8); attention and mental processing speed (*MAPTn* = 8; *GRNn* = 3; *C9orf72n* = 8); executive functioning (*MAPTn* = 9; *GRNn* = 2; *C9orf72n* = 8); working memory (*MAPTn* = 5; *GRNn* = 2; *C9orf72n* = 6); memory learning (*MAPT* n = 7; *GRNn* = 3; *C9orf72n* = 8); memory recall (*MAPTn* = 7; *GRNn* = 3; C*9orf72n* = 7); visuoconstruction (*MAPTn* = 6; *GRNn* = 2; *C9orf72n* = 7)

We explored individual trajectories of cognitive decline in a subset of patients (*n* = 27) that underwent multiple neuropsychological assessments (Fig. 1; Online Resource). Overall, *GRN* mutation carriers (*n* = 3) showed the largest decline of all mutation carrier groups in the first year after diagnosis. Specifically, these patients declined most on attention, mental processing speed and memory. *MAPT* mutation carriers (*n* = 13) performed at an intermediate level between *GRN* and *C9orf72* mutation carriers (*n* = 11) on all tests, but did not seem to decline more profoundly on a specific cognitive domain compared to other domains. *C9orf72* repeat expansion carriers showed the most stable trajectories with minimal decline on most domains. *MAPT* mutation carriers performed lower and declined most on the BNT, whereas *GRN* mutation carriers declined most on the TMT A and B (Online Resource). Although the RAVLT showed lower performance in *MAPT* mutation carriers, a steeper decline over time was seen in *GRN* mutation carriers (Online Resource).

## Discussion

This study demonstrated gene-specific neuropsychological profiles within the clinical phenotype of bvFTD. The three mutation carrier groups were impaired on all cognitive domains compared to non-carriers, except for working memory and visuoconstruction. Interestingly, patients with bvFTD could be differentiated according to genetic mutation both on cognitive domain level and on neuropsychological test level. Attention and mental processing speed, as well as executive functioning differentiated *GRN* from *MAPT* and *C9orf72*, and memory recall deficits seemed a distinctive feature of *MAPT*. Executive functioning was most sensitive to differentiate *GRN* mutation carriers from non-carriers, whereas language was most sensitive to differentiate *MAPT* and *C9orf72* mutation carriers from non-carriers. Within-individual trajectories indicated a more rapid decline on attention and memory in *GRN* mutation carriers and confrontation naming in *MAPT* in the first year after diagnosis, whereas *C9orf72* repeat expansion carriers remained relatively stable on all domains.

Studies in both presymptomatic [14] and symptomatic *GRN* mutation carriers [21] have shown impairment and/or decline in attention and mental processing speed. An explanation for this decline (in fronto-subcortical functions) is the extensive subcortical white matter lesions that are regularly seen in *GRN* mutation carriers [35]. The subcortical structures of the brain are thought to be especially important for information processing speed, and lesions in these structures have, therefore, been primarily associated with difficulties in attention and mental processing speed as well as executive functioning [5]. Interestingly, multiple neuroimaging studies have shown that *GRN* mutations are associated with marked asymmetrical cortical atrophy, with either left or right sided predominance [21]. It has been argued that these differences in patterns of neurodegeneration can be reflected in different cognitive profiles [21]. Additional analyses showed that *GRN* patients with more pronounced left-sided atrophy performed worse on language than patients with more pronounced right-sided atrophy. This is unsurprising given that language processing is strongly left lateralized [11]. There were no other cognitive differences between patients with either primarily left-sided, right-sided or bilateral atrophy. Due to small sample sizes groups were not stratified according to the pattern of neurodegeneration in the main part of the analyses, but grouping them together may have influenced results (particularly language performance) for this group.

Within-individual trajectories in *GRN* mutation carriers showed a rapid decline on all cognitive domains in the first year after diagnosis. This rapid cognitive decline in *GRN* mutation carriers is also partially reflected by the finding that the majority of 17 cases that did not undergo repeated neuropsychological assessment were too severely cognitively impaired for testing at follow-up (i.e., residing in nursing home or unable to complete multiple neuropsychological tests at baseline). This finding is confirmed by other studies reporting a shorter disease duration [3] and more rapid changes following symptom onset in *GRN* mutation carriers [15]. The most profound decline was seen on attention/mental processing speed and memory. Memory problems have previously been described in *GRN* as a symptom characterizing progressed disease stages [16], although it could also be associated with the profound impairment in attention/mental processing speed [31].

*MAPT* mutation carriers were the only group impaired on both immediate and delayed recall at baseline, whereas *GRN* and *C9orf72* mutation carriers were only impaired on immediate recall. This is in line with a previous study by Jiskoot et al. [16] that demonstrated significant decline on the RAVLT recall test in the presymptomatic stage of *MAPT* mutation carriers, with a further decline in participants that converted to symptomatic FTD during follow-up. This is further corroborated by the finding that the RBMT direct and delayed recall trials were most sensitive to differentiate *MAPT* mutation carriers from non-carriers. Memory impairment has previously been described as a prominent symptom in patients with a *MAPT* mutation, possibly due to anteromedial temporal lobe atrophy that is often seen in *MAPT *[28]. This is an area that has been associated with defects in memory storage and consolidation, as is the case in for instance Alzheimer’s disease [34]. Another hypothesis that has been suggested is that memory deficits in bvFTD are a consequence of executive dysfunctioning (i.e., poor organization and lack of efficient learning strategies) due to prefrontal atrophy [14]. This suggests that memory impairment differs between bvFTD patients depending on the underlying mutation and thus atrophy pattern, with *MAPT* mutation carriers demonstrating a “pure” memory impairment resulting in lower performance on both immediate and delayed recall, whereas the immediate recall impairment in *C9orf72* and *GRN* mutation carriers are potentially a consequence of prefrontal and thus dysexecutive impairment, with relatively spared delayed recall performance.

In contrast to the findings of previous studies, *MAPT* mutation carriers in the current cohort did not show worse semantic functioning compared to *GRN* and *C9orf72* mutation carriers [26]. This discrepancy might be explained by the use of estimated 60-item versions of the BNT, a “semantic” confrontation naming test, from 15-item BNT administrations. A validation study has shown that the 15-item BNT has lower sensitivity and diagnostic accuracy compared to the 60-item version of the BNT [12]. Another explanation might be that the nature of naming errors differed in each genetic variant. *MAPT* mutation carriers were relatively more impaired on BNT and semantic fluency compared to letter fluency, whereas *GRN* performed equally impaired on all language tests, suggesting different underlying mechanisms (e.g., semantic problems versus dysexecutive control) (e.g., [36]. We included all fluency tasks in the language domain, but it has been previously demonstrated that fluency also involves other cognitive functions such as executive functioning and semantic memory. [36, 40]. Furthermore, within-individual trajectories showed that *MAPT* mutation carriers declined most on the BNT. It might also be possible that the occurrence of semantic impairments become more prominent in *MAPT* in a later stage of the disease [28], as anterior medial temporal lobe atrophy progresses, an area that has been linked to semantic naming errors in for instance Alzheimer’s disease [2] and is known to also deteriorate bilaterally in patients with a *MAPT* mutation [28].

Patients with a *C9orf72* repeat expansion showed a widespread and non-progressive pattern of cognitive impairment in language, attention/mental processing speed, executive functioning and immediate recall and no distinctive cognitive impairment compared to *GRN* and *MAPT* mutation carriers. This cognitive profile is corroborated by studies indicating that the neurodegenerative process associated with the *C9orf72* repeat expansion is also widespread, with degeneration in the frontal and temporal cortices but also subcortical and cerebellar regions [24]. It has been demonstrated that the first brain changes start to emerge already in early adulthood but do not evolve, suggesting that they reflect an abnormal neurodevelopmental trajectory rather than early neurodegeneration [22]. This possibly also explains the slowly progressive bvFTD cases that have, in particular, been associated with the *C9orf72* repeat expansion [19], and were also seen in our within-individual trajectories. One theory suggests that neuropsychiatric symptoms represent the clinical prodrome of bvFTD in *C9orf72* repeat expansion carriers, with cognitive deterioration occurring only in progressed disease stages [22].

Overall, results show that it is possible to distinguish between genetic variants of bvFTD using specific neuropsychological domains and tests. This enables the identification of sensitive tests for signaling disease onset and predicting disease progression in clinical practice and could inform future therapeutic trials in selecting clinical endpoints to monitor treatment response. The former could be helpful in providing psycho-education and counseling to the patient and caregiver on the expected clinical presentation and disease course. Moreover, selection of the most sensitive tests per genetic defect enables shortening of the neuropsychological test battery thereby relieving patient burden [16]. Executive tasks, such as letter fluency, and tasks for attention and mental processing speed, such as TMT and SCWT, were most sensitive to detect *GRN-*associated FTD, whereas memory recall deficits seem a promising marker in *MAPT-*associated FTD. There does not appear to be a specific cognitive domain/test that can differentiate *C9orf72* from other genetic variants, possibly due to the widespread neurodegenerative process affecting multiple cognitive domains equally and the slow progression. Importantly, though this study shows statistical differences between mutation carriers groups, there is still a considerable percentage of overlap on all cognitive domains and tests, with letter fluency having the lowest %OL between groups (26.9–34.7%OL). In addition, *GRN* mutation carriers performed relatively worse on all cognitive tests, possibly due to an altogether greater disease severity. Although we corrected for disease duration in our analyses, a reliable instrument for disease severity (e.g., FTD-CDR) was lacking. Similarly, exploratory longitudinal descriptions provided valuable information on rate of cognitive decline with indeed rapid cognitive decline in *GRN*, but slow progression in *C9orf72* and *MAPT* showing intermediate decline. Taking this together, results should be interpreted carefully. We report several clear differences between genetic mutations, but given the relatively wide range of cognitive impairments (i.e., multiple domains affected) found in our patient sample, and the high percentage of overlap between patient groups, it remains challenging to identify a gene-specific cognitive profile in individual patients. Our results should be viewed as guidance for selecting clinical endpoints in future therapeutic trials rather than recommendations for the ‘best’ neuropsychological test to be used.

The current longitudinal descriptions should be carefully interpreted as sample sizes are small. Further limitations are the changes in neuropsychological test protocol with different tests and test versions used over time during the extended time of the study. In addition, we did not include tasks measuring social cognition, a key feature in diagnosing bvFTD [27], as social cognitive tasks were only added to the standard neuropsychological assessment in our memory clinic since 2012, resulting in too small sample sizes for the current analysis (*n* < 10 in each group). A more clear dissociation between attention/mental processing speed and executive functioning tasks could have been made by analyzing the inter-relationship between TMT A and B, and SCWT II and III. However, for several patients who were unable to complete the test, we truncated the score to 300 s. These patients typically already had a much higher completion time on TMT A or SCWT II, and calculating the ratio would, therefore, have resulted in optimizing the ratio-score specifically for those patients that were too cognitively impaired to complete the test.

This study presents a large cohort of genetic bvFTD patients, including three major genetic causes of FTD, with unique neuropsychological data covering a wide variety of tests in seven cognitive domains. We provide evidence of gene-specific cognitive profiles within patients with bvFTD and provide recommendations for the use of specific tests to assess gene-specific clinical onset and disease progression. This is important information for future clinical trials targeting specific pathologies as clinical endpoints to monitor treatment response are increasingly urgent.

## Electronic supplementary material

Below is the link to the electronic supplementary material.

Supplementary file1 (PDF 410 kb)
