# Correction to: Cognitive profiles discriminate between genetic variants of behavioral frontotemporal dementia

**DOI:** 10.1007/s00415-020-09784-6

**Published:** 2020-03-26

**Authors:** J. M. Poos, L. C. Jiskoot, S. M. J. Leijdesdorff, H. Seelaar, J. L. Panman, E. L. van der Ende, M. O. Mol, L. H. H. Meeter, Y. A. L. Pijnenburg, L. Donker Kaat, F. J. de Jong, J. C. van Swieten, J. M. Papma, E. van den Berg

**Affiliations:** 1 Department of Neurology, Alzheimer Center, Erasmus MC University Medical Center, Dr. Molewaterplein 40, 3000 CA Rotterdam, The Netherlands; 2 Department of Radiology, Leiden University Medical Center, Leiden, The Netherlands; 3 Dementia Research Center, University College London, London, UK; 4 Department of Psychiatry and Psychology, Maastricht University, Maastricht, The Netherlands; 5 Department of Neurology, Alzheimer Center, Location VU University Medical CenterAmsterdam Neuroscience, Amsterdam University Medical Center, Amsterdam, The Netherlands; 6 Department of Clinical Genetics, Erasmus MC University Medical Center, Rotterdam, The Netherlands

## Correction to: Journal of Neurology 10.1007/s00415-020-09738-y

The original version of this article unfortunately contained a mistake. The presentation of Table 1 was incorrect.

The original article has been corrected.

The corrected Table 1 is given below.

**Table 1:** Demographic features

	*MAPT* mutation carriers (*n* = 29)	*GRN* mutation carriers (*n* = 20)	*C9orf72* mutation carriers (*n* = 31)	Non-carriers (*n* = 24)	*p* Value	Group differences
Age at baseline, y	52.6 ± 5.5	60.4 ± 7.4	62.1 ± 9.1	56.1 ± 5.7	< 0.01	*MAPT* < *GRN* = *C9orf72* NC < *C9orf72*
Sex (% female)	10 (34.5%)	12 (57.1%)	13 (41.9%)	11 (45.8%)	0.6	n.s
Educational level ^a^ (median (IQR))	5 (2)	5 (2)	5 (2)	5 (0)	0.8	n.s
Disease duration, y	1.4 ± 2.0	1.0 ± 1.1	3.1 ± 2.7	NA	< 0.01	*MAPT* = *GRN* < *C9orf72*
Subtype dis—apa—ster	9—15—5	6—14—0	6—21—3	NA	0.3	n.s
MMSE	25.9 ± 2.9	22.5 ± 6.3	26.5 ± 2.7	29.3 ± 0.8	< 0.01	*GRN* < *MAPT* < NC *GRN* < *C9orf72*
FAB	14.7 ± 3.2	10.0 ± 4.7	13.9 ± 3.4	16.1 ± 1.7	< 0.01	*GRN* < *MAPT* = NC

Values indicate mean ± SD or n (%) unless otherwise specified

*MAPT* microtubule-associated protein tau, *GRN* progranulin, *C9orf72* chromosome 9 open reading frame 72, *NC* non-carriers, *dis* disinhibited, *apa* apathetic, *ster* stereotypic, *MMSE* Mini-Mental State Examination, *FAB* Frontal Assessment Battery, *n.s* not significant

^a^Verhage Dutch educational system categorized into levels from 1 = less than 6 years of primary education to 7 = academic schooling

