# Supplementary material for: Cognitive profiles discriminate between genetic variants of behavioral frontotemporal dementia
Source: J Neurol. 2020 Feb 12;267(6):1603–12. doi: 10.1007/s00415-020-09738-y (PMC7293665; doi:10.1007/s00415-020-09738-y)
Supplement: Supplementary file 1 — Supplementary file1 (PDF 410 kb) [file 415_2020_9738_MOESM1_ESM.pdf]

Supplementary material

Article title: Cognitive profiles discriminate between genetic variants of behavioral frontotemporal dementia

Journal name: Journal of Neurology

Author names and affiliation:

Poos, J.M. MSc[12], Jiskoot, L.C. PhD[13], Leijdesdorff, S.M.J. MSc.[4], Seelaar, H. MD PhD[1], Panman, J.L. MSc. [12] , van der Ende, E.L MD[1], Mol, M.O. MD[1], Meeter, L.H.H. MD PhD[1], Pijnenburg, Y.A.L. MD PhD[5], Donker Kaat, L. MD PhD[16], de Jong, F.J. MD PhD[1], van Swieten, J.C. MD PhD[1], Papma, J.M. PhD[1], van den Berg, E. PhD[1]

[1]Alzheimer Center and Department of Neurology, Erasmus MC University Medical Center, Rotterdam, the Netherlands

[2]Department of Radiology, Leiden University Medical Center, Leiden, the Netherlands

[3]Dementia Research Center, University College London, London, UK

[4]Department of Psychiatry and Psychology, Maastricht University, Maastricht, the Netherlands

[5]Alzheimer center, Department of Neurology, Amsterdam University Medical Center, location VU University Medical Center, Amsterdam Neuroscience, Amsterdam, the Netherlands

[6] Department of Clinical Genetics, Erasmus MC University Medical Center, Rotterdam, The Netherlands

E-mail address corresponding author: j.m.poos@erasmusms.nl

Table 1. Differences between *GRN* patients with predominant right-sided, left-sided or generalized atrophy on seven cognitive domains.

**Domain**	**Left**	*n*	**Right**	*n*	**Generalized**	*n*	*p* Value
**Language**	-3.3 ± 1.2	10	-1.0 ± 0.7	4	-2.1 ± 0.9	4	0.02
**Attention and mental processing speed**	-6.6 ± 4.2	9	-0.7 ± 0.7	4	-3.6 ± 3.8	3	0.2
**Executive functioning**	-6.2 ± 2.0	9	-2.2 ± 1.5	4	-5.1 ± 2.2	4	0.09
**Memory - learning**	-5.6 ± 6.5	9	-3.5 ± 5.2	4	-7.0 ± 9.3	3	0.8
**Memory - recall**	-1.1 ± 1.6	7	-0.3 ± 2.2	4	-1.0 ± 1.7	3	0.8
**Working memory**	-2.1 ± 1.0	4	-0.1 ± 2.7	4	-	0	0.5
**Visuoconstruction**	-1.2 ± 1.7	9	-0.3 ± 0.8	4	-0.9 ± 2.0	3	0.4

Values indicate mean ± SD. The *p* values constitute interaction terms of univariate analyses of covariance (corrected for age and education).

Fig. 1 Within-individual trajectories on neuropsychological tests

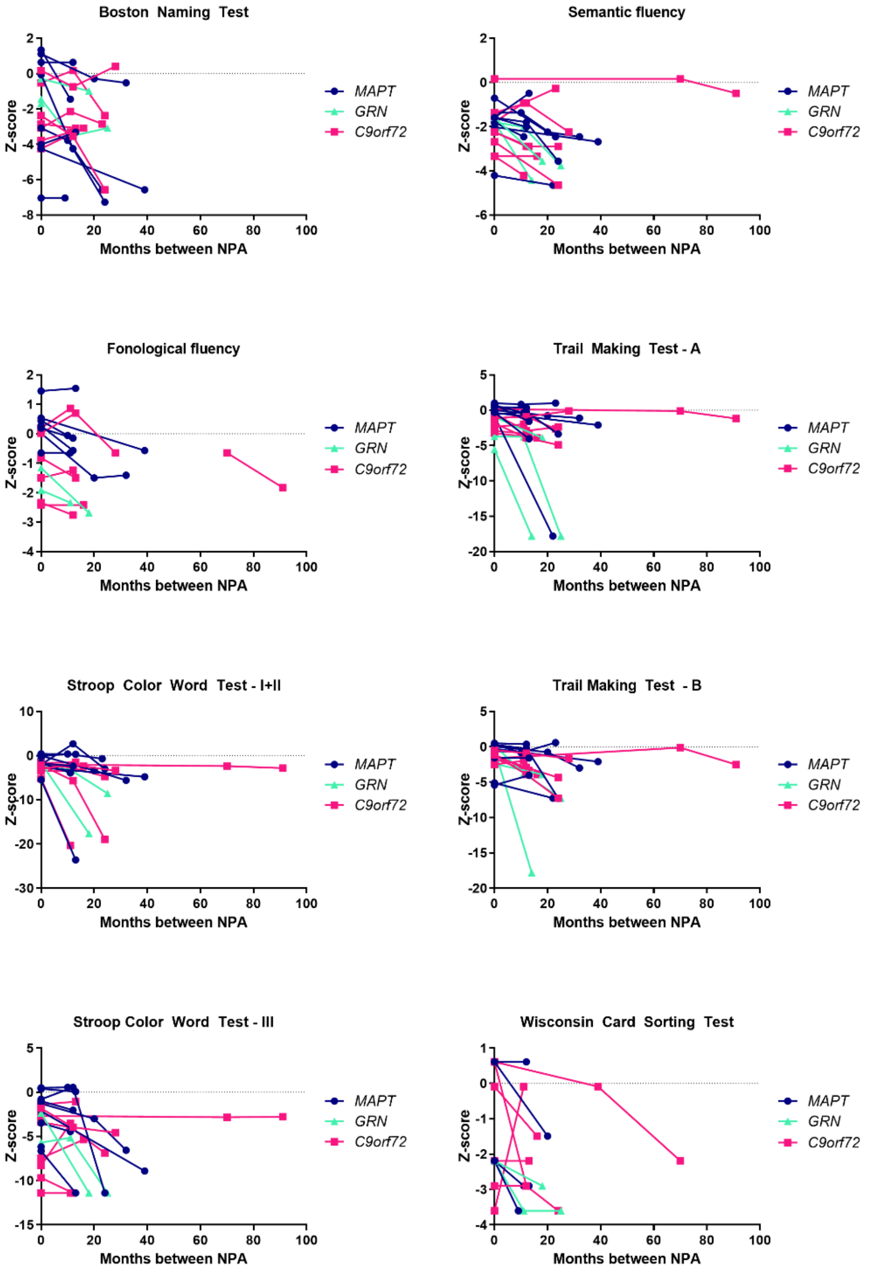

Abbreviations: NPA = neuropsychological assessment; *MAPT* = microtubule-associated protein tau; *GRN* = progranulin; *C9orf72* = chromosome 9 open reading frame 72. Raw data for each neuropsychological test were first converted to z-scores by standardization to the baseline data of healthy controls. Composite cognitive domain scores were calculated. Each subplot presents the trajectory on a specific cognitive domain. Data are available in: Boston Naming Test (*MAPT* n=8; *GRN* n=2; *C9orf72* n=7); semantic fluency (*MAPT* n=8; *GRN* n=3; *C9orf72* n= 8); letter fluency (*MAPT* n=7; *GRN* n=2; *C9orf72* n=6); Trail Making Test - A (*MAPT* n=9; *GRN* n=3; *C9orf72* n= 8); Stroop Color Word Test I+II (*MAPT* n=8; *GRN n*=2; *C9orf72* n=8); Trail Making Test - B (*MAPT* n=8; *GRN* n=3; *C9orf72* n=9); Stroop Color Word Test III (*MAPT* n= 8; *GRN* n=2; *C9orf72* n=8); Wisconsin Card Sorting Test (*MAPT* n=4; *GRN* n=2; *C9orf72* n=6).

Fig. 2 Within-individual trajectories on neuropsychological tests.

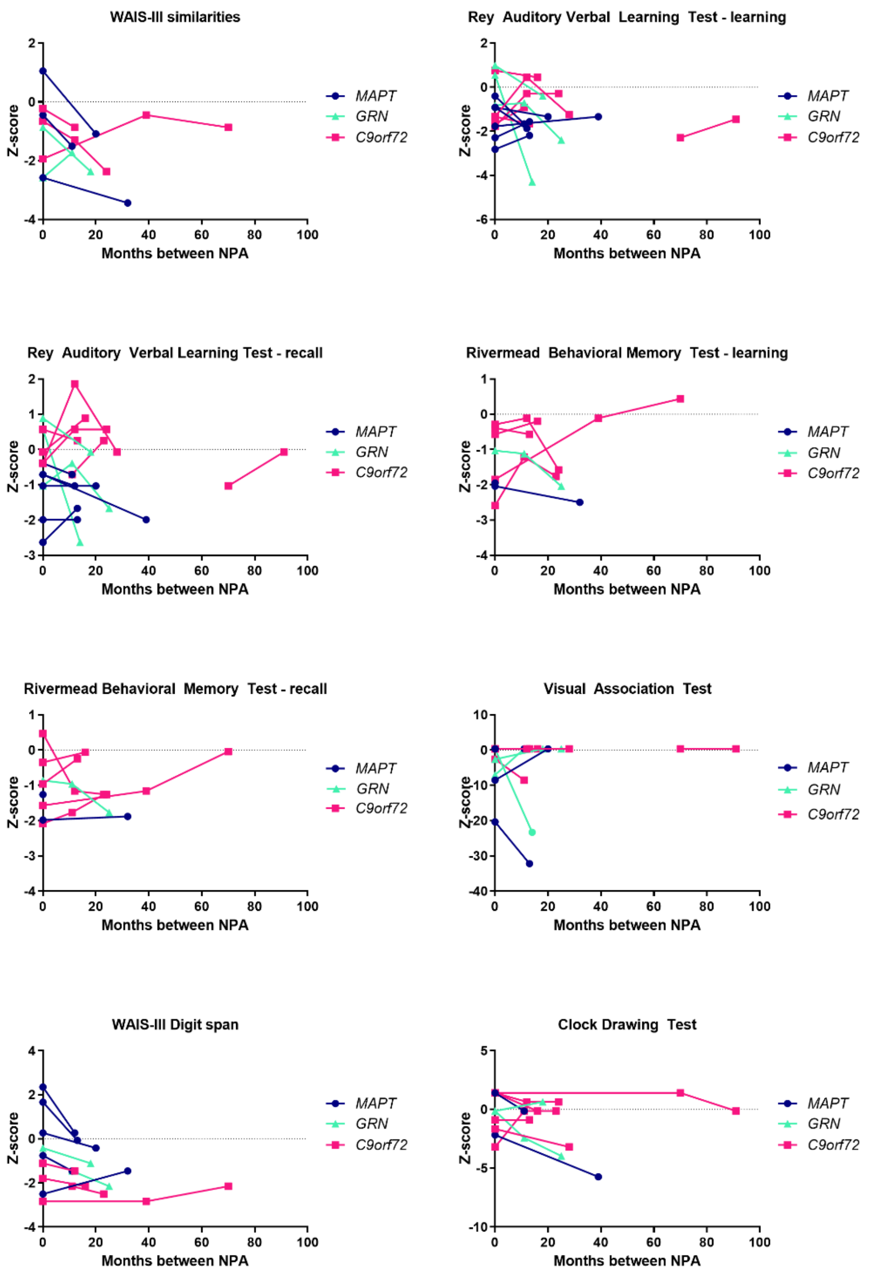

Abbreviations: NPA = neuropsychological assessment; *MAPT* = microtubule-associated protein tau; *GRN* = progranulin; *C9orf72* = chromosome 9 open reading frame 72. Raw data for each neuropsychological test were first converted to z-scores by standardization to the baseline data of healthy controls. Composite cognitive domain scores were calculated. Each subplot presents the trajectory on a specific cognitive domain. Data are available in: WAIS-III Similarities (*MAPT* n= 3; *GRN* n=2; *C9orf72* n= 3); Rey Auditory Verbal Learning Test – learning (*MAPT* n= 6; *GRN* n=3; *C9orf72* n=6); Rey Auditory Verbal Learning – recall (*MAPT* n=6; *GRN* n=3; *C9orf72* n=6); Rivermead Behavioral Memory Test - learning (*MAPT* n=1; *GRN* n=1; *C9orf72* n=6); Rivermead Behavioral Memory Test - recall (*MAPT* n=5; *GRN* n=1; *C9orf72* n= 6); Visual Association Test (*MAPT* n= 3; *GRN* n=3; *C9orf72* n=3); WAIS-III Digit Span (*MAPT* n= 5; *GRN* n=2; *C9orf72* n=4); Clock Drawing Test (*MAPT* n=2; *GRN* n=2; *C9orf72* n=6).
